# Comprehensive Genome‐Wide Analysis of Shared Genetic Factors in Gastrointestinal and Neurodegenerative Diseases

**DOI:** 10.1002/brb3.71029

**Published:** 2025-11-23

**Authors:** Yan Jiang, Yuxiang Zhang, Lei Ma, Chao Li

**Affiliations:** ^1^ Laboratory Medicine Diagnostic Centre, The First Affiliated Hospital Xinjiang Medical University Urumqi Xinjiang China; ^2^ Department Laboratory, The Six Affiliated Hospital Xinjiang Medical University Urumqi Xinjiang China

**Keywords:** amyotrophic lateral sclerosis, gastrointestinal diseases, genetic relatedness, genome‐wide association study, Mendelian randomization, neurodegenerative diseases

## Abstract

**Background:**

This study investigates the shared genetic basis between gastrointestinal (GI) diseases and neurodegenerative diseases (ND) using genome‐wide association study (GWAS) data and statistical genetic methods.

**Methods:**

GWAS data, primarily from European populations, covered four types of GI diseases and three types of ND. Genetic correlations were assessed using Linkage Disequilibrium Score Regression (LDSC), High‐Definition Likelihood (HDL), and Local Analysis of Covariant Annotation (LAVA). Pleiotropic and functional genetic overlaps were explored using the Genomic Partitioning Approach (GPA), pleiotropic analysis under the composite null hypothesis (PLACO), and Functional Mapping and Annotation of Genetic Associations (FUMA). Multi‐marker analysis of genomic annotation (MAGMA) was used for biological annotation and enrichment analysis, while summary data‐based Mendelian randomization (SMR) analysis linked pleiotropic genes with gene expression. Two‐sample Mendelian randomization (TSMR) investigated potential causal relationships.

**Results:**

Significant genetic correlations and shared genetic features were identified. The research identified 1,457 pleiotropic single nucleotide variants(SNVs) distributed across 47 chromosomal regions and 74pleiotropic genes, predominantly involved in pathways related tosignal transduction, amyloid regulation, and lipid metabolism. Nine colocalization loci (PPH4 > 0.8) were identified, while SMR analysis linked 26 pleiotropic genes to disease expression levels. Key genes, including *EP300*, *CHRNB1*, *KNOP1*, *P2RY14*, and *POLR2A*, were significantly associated with both disease types. Drug‐gene interaction analysis highlighted 8 genes with drug targets, among which *EP300*, *PRKCB*, *VKORC1*, and *CHRNB1* were found to anchor enriched pathways including purinergic signaling, amyloid/protein aggregation, and cholesterol/lipoprotein transport. Mendelian randomization corroborated possible causal association.

**Conclusion:**

This study confirms a shared genetic basis between GI and ND, emphasizing the gut‐brain axis in their etiology. These findings offer clues about shared pathways, potential therapeutic targets, and future research directions.

## Graphical abstract

GI and ND GWAS and blood eQTL data were analyzed using LDSC, HDL, LAVA, GPA, PLACO, FUMA, MAGMA, SMR, HEIDI, and two‐sample MR to assess genetic correlations, pleiotropy, functional annotation, shared gene expression, and causal relationships.

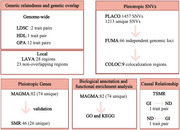

## 1 Introduction

Gastrointestinal (GI) diseases constitute a spectrum of disorders that have a significant impact on health systems and society. These diseases, particularly functional GI disorders such as irritable bowel syndrome (IBS) and functional dyspepsia, affect up to 40% of the population, but their symptoms are often difficult to explain by organic factors. At the same time, neurodegenerative diseases (ND) pose a major challenge, not only affecting important central nervous system functions but also leading to persistent intestinal dysfunction. This situation suggests a complex bidirectional influence between the CNS and intestinal neurons (Black et al. 2020, Mossa et al. 2023, Singh et al. 2021).

Bowel disorders such as IBS are closely associated with the development and pathology of ND and neuropsychiatric disorders. IBS, a chronic functional GI disorder, significantly impacts life quality. It's known for “leaky gut” syndrome, where the intestinal blood barrier's disruption leads to widespread inflammation (El‐Hakim et al. 2022). The same is true for inflammatory bowel disease (IBD). Similarly, the link between IBD and ND is of increasing interest. Research has revealed that individuals with IBD have a heightened risk of Alzheimer's (AD) and Parkinson's diseases (PD). This connection, confirmed through multiple meta‐analyses, underscores a significant link between GI disorders and ND (Szandruk‐Bender et al. 2022).

ND affects more than 6 million people in the United States. Among them, diseases like amyotrophic lateral sclerosis (ALS) have a profound impact on patients and their caregivers. These diseases often progress rapidly and can significantly shorten a patient's life expectancy (Mossa et al. 2023). For caregivers, this rapid disease progression presents a significant psychological and quality of life burden. The incidence of ND is rising rapidly as the global population is aging. By 2050, the number of cases of diseases such as dementia is expected to increase significantly (Zheng and Chen 2022).

The gut‐brain axis (GBA) forms a critical communication network between the GI tract and the central nervous system (CNS), encompassing neural, immune, and endocrine pathways. Imbalances in this axis are associated with various ND conditions, including AD, PD, and ALS (Zheng et al. 2023).

The neuroimmune communication within the GBA is crucial for such interactions. Understanding how gut microbiota influences the neuroimmune system is essential in unraveling the underlying mechanisms of ND. This new understanding has led to a shift in research focus towards the development of new therapeutic strategies targeting the central nervous system, utilizing insights into the gut microbiota and the GBA (Zheng et al. 2023).

In addition, microbe‐host interaction studies have emphasized the impact of the gut microbiota on the enteric nervous system, thus further elucidating the complex links between the GI system and ND processes (Hyland and Cryan 2016).

In this research, varieties of techniques were utilized to assess the genetic relationships and shared genetic aspects between GI and ND. Initially, the research was based on publicly available datasets, including genome‐wide association study (GWAS) data for four GI disorders and three ND, primarily from European populations, to minimize the impact of ethnic differences. This research applied Linkage Disequilibrium Score Regression (LDSC) and High‐Definition Likelihood (HDL) techniques for evaluating genetic correlations between 12 pairs of traits. Additionally, Genetic Analysis Incorporating Pleiotropy and Annotation (GPA) were employed to deeply investigate the genetic overlap between different traits. For identifying pleiotropic loci and conducting co‐localization analyses, the study used Pleiotropy Analysis under Compound Null Hypothesis (PLACO) and MAGMA‐based genome‐level expression and functional enrichment analysis. Last, potential causal relationships between GI and ND were explored using two‐sample Mendelian randomization (TSMR) analysis.

## 2 Methods

**FIGURE 1:**
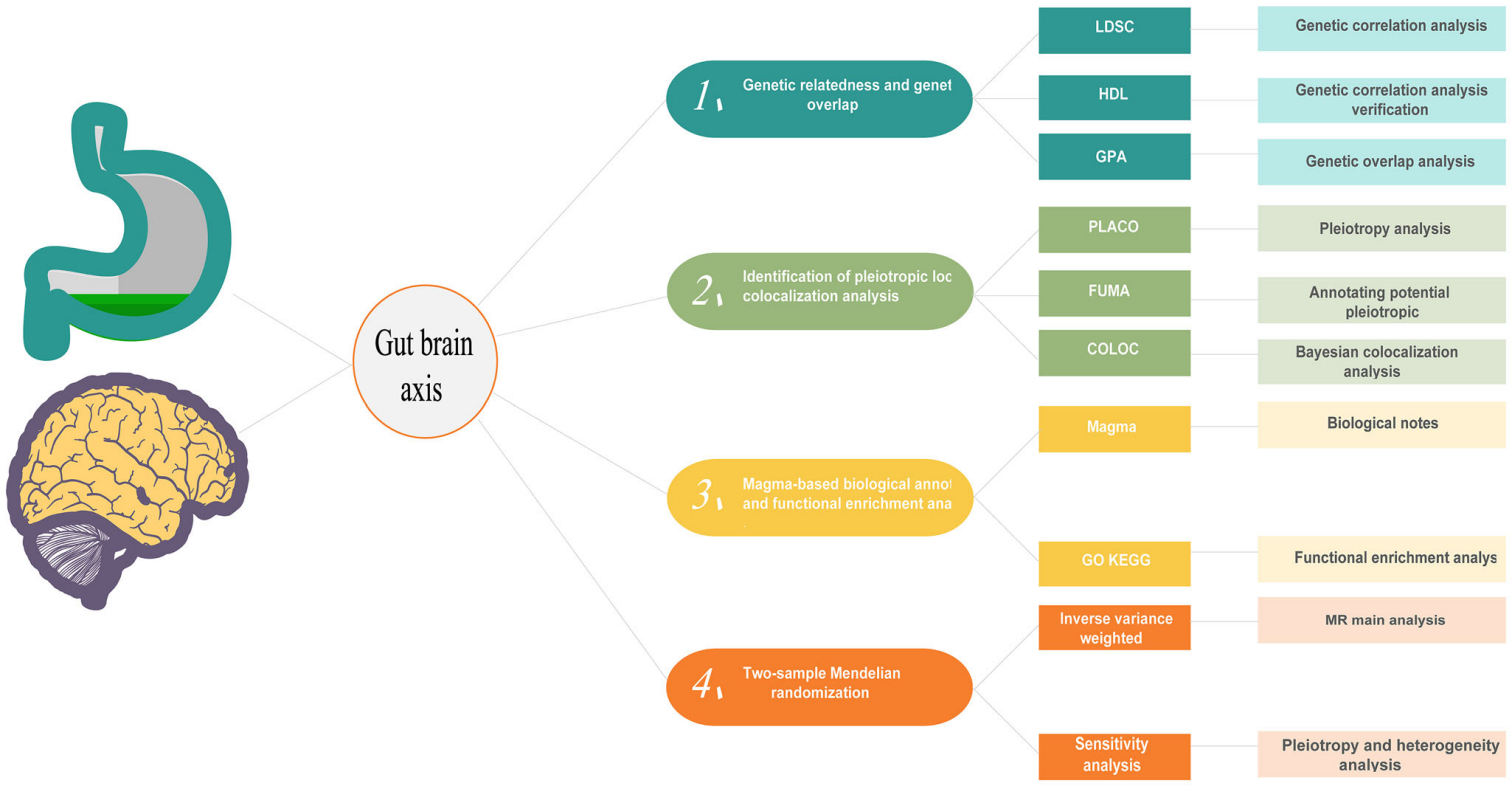
Overview of the methods used in this study. (Abbreviations: COLOC, co‐localization analysis; FUMA, Functional Mapping and Annotation of Genetic Associations; GPA, Genetic Analysis Incorporating Pleiotropy and Annotation; HDL, High‐Definition Likelihood; LDSC, Linkage Disequilibrium Score Regression; MAGMA, GenoMic Annotation; PLACO, Pleiotropy Analysis under Compound Null Hypothesis.).

**FIGURE 2:**
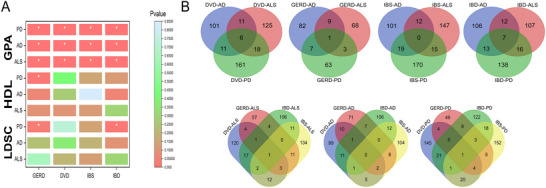
Genetic correlation and genetic overlap diagram. **(A)** Genetic correlation chart. *indicates a *p*‐value less than 0.05 and **(B)** Diagram of locus overlaps with significant local genetic correlation p‐values.

**FIGURE 3:**
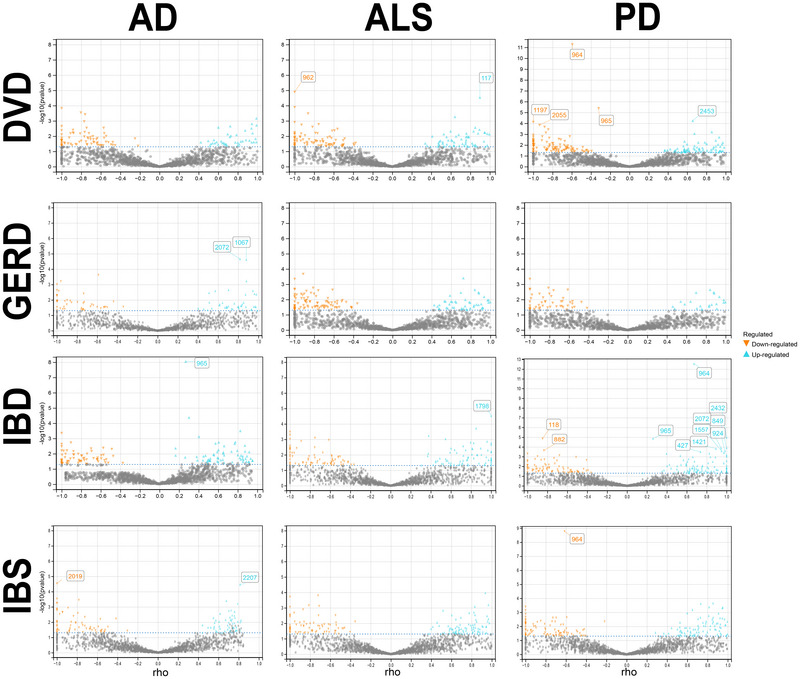
Volcano plot of local genetic correlation between GI and ND. Orange dots represent significant negative correlation, and blue dots represent significant positive correlation (*p*‐value < 0.05). Points with numbers are still significant after FDR correction (P_FDR_ < 0.05).

**FIGURE 4:**
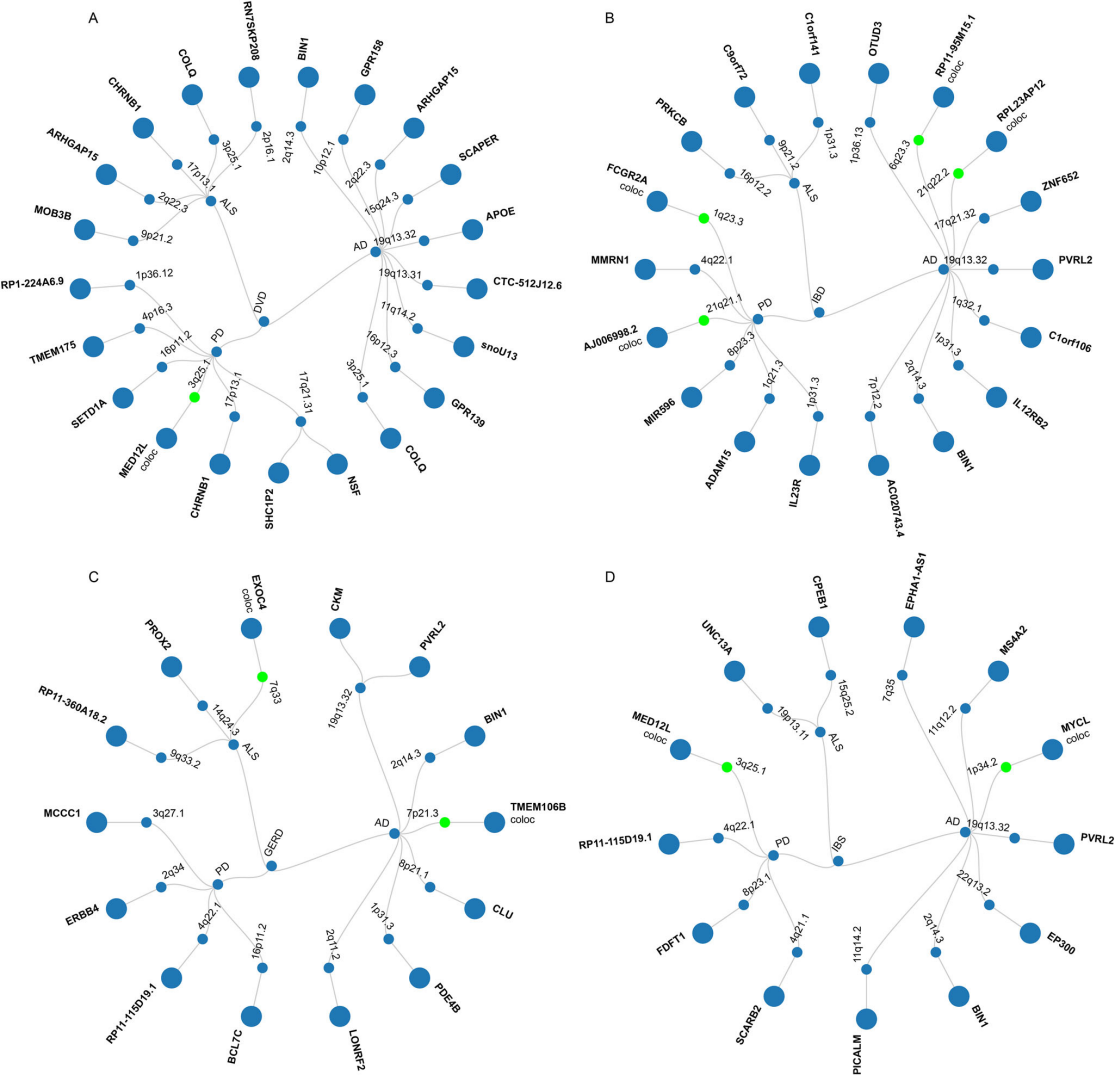
Circular dendrogram of 12 trait pairs—This visualization illustrates pleiotropic loci shared between GI and ND. Green highlights indicate regions with a colocalization coefficient greater than 0.7. Genes mapped via FUMA positional mapping are shown, while “coloc” denotes genes near colocalized loci. Panels: A: Pleiotropic loci between DVD and three ND traits; B: Pleiotropic loci between IBD and three ND traits; C: Pleiotropic loci between GERD and three ND traits; D: Pleiotropic loci between IBS and three ND traits.

**FIGURE 5:**
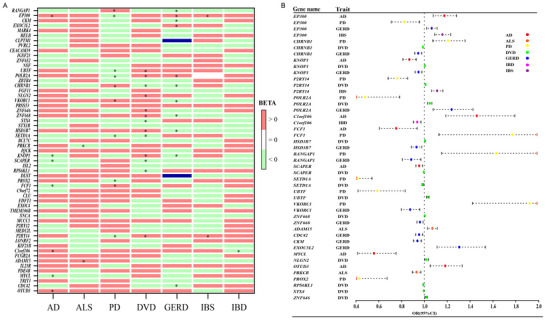
Identification of Pleiotropic Genes. **(A)** MAGMA‐based analysis of pleiotropic gene eQTLs and SMR analysis heatmap for GI and ND. *indicates significant results (P_fdr_ < 0.05). **(B)** Forest plot of SMR analysis for pleiotropic gene eQTLs based on MAGMA analysis in GI and ND.

**FIGURE 6:**
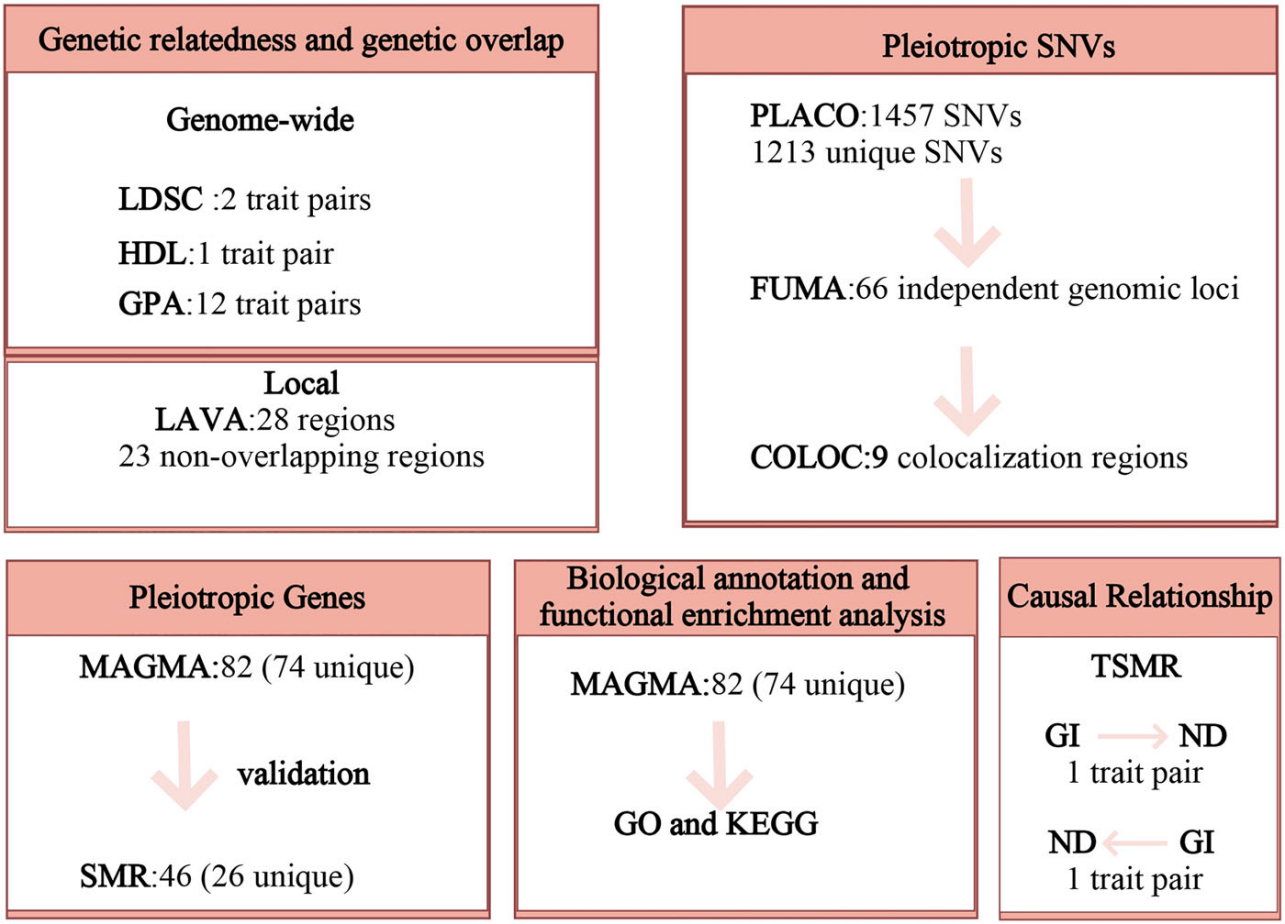
Flowchart of the study workflow. Publicly available GWAS and blood eQTL data for GI and ND were collected. Genetic correlations and overlaps were assessed using LDSC, HDL, LAVA, and GPA. Significant trait pairs underwent pleiotropic locus identification via PLACO and functional annotation with FUMA and MAGMA. Shared gene expression was analyzed using SMR and HEIDI, and causal relationships were evaluated with TSMR.

The research process is shown in (Figures 1, 2, 3, 4, 5, 6)

### 2.1 Data Sources

The GWAS data used in this study come from publicly available datasets from IEU and GWAS Catalog, including four GI diseases (IBD (Glanville et al. 2021), IBS (Eijsbouts et al. 2021), diverticular disease (DVD) (Schafmayer et al. 2019), and gastroesophageal reflux disease (GERD) (Ong et al. 2022)) and three ND (ALS (van Rheenen et al. 2021), AD excluding UK Biobank and 23andMe data (Wightman et al. 2021), and PD from the International Parkinson's Disease Genomics Consortium) (Supplementary Table S1). Blood eQTLs were sourced from the eQTLGen Consortium, which encompasses information on 10,317 single nucleotide polymorphisms (SNPs) associated with traits from 31,684 individuals (Võsa et al. 2021). The total sample size of the GWAS data exceeded 100,000, primarily from European populations to minimize ethnic differences. AD data SNPs were imputed using the hg19 genome version from https://hgdownload.soe.ucsc.edu/downloads.html. All GWAS analyses in this study were based on the human genome reference sequence GRCh37 (also known as hg19), chosen for its widespread support in genetic databases and analytical tools, ensuring accuracy and consistency in comparative research. In this study, the SNVs involved were excluded from all analyses except for LAVA in the MHC region (chromosome 6: 25–35 MB).

### 2.2 Genetic Correlation and Genetic Overlap

In this study, we applied Linkage Disequilibrium Score Regression (LDSC), High‐Definition Likelihood (HDL), and Local Analysis of Variant Annotation (LAVA) methods to assess the genome‐wide genetic correlations among 12 pairs of traits between four GI diseases and three ND (Werme et al. 2022). Quantifying the contribution of each element by examining the relationship between the test statistic and LDSC allowed determining whether the different traits share a common genetic basis (Bulik‐Sullivan et al. 2015). As a powerful method for estimating heritability and genetic correlations based on GWAS summary statistics, the LDSC can be used to distinguish between true polygenicity and mixed biases (e.g., population stratification and recessive associations) (Xu et al. 2022). The initial analysis phase involves applying cross‐trait LDSC (https://github.com/bulik/ldsc) and HDL (Ning et al. 2020) methods to estimate genetic correlations. Compared to LDSC, HDL comprehensively accounts for genome‐wide linkage disequilibrium (LD), thereby significantly improving the precision of the estimation. Local Analysis of Variant Annotation estimated local genetic correlations in 2495 genome‐wide regions. To adjust for multiple comparisons, the Benjamini–Hochberg method was applied across all pairwise comparisons.

Genetic correlations only reveal genome‐wide associations; hence, Genetic Analysis Incorporating Pleiotropy and Annotation (GPA) methods were further employed to explore comprehensive genetic overlaps among different traits (Gong et al. 2023). In GPA analysis, four models (M00, M10, M01, M11) categorized SNPs into four groups, assessing the overall genetic overlap's statistical significance through likelihood ratio tests (LRT). This method allowed a more thorough understanding of genetic interactions between traits and provided richer genetic background information for the study. Significance levels were set at *p* < 4.17 × 10^−3^ (0.05/12) after Bonferroni correction. *p*‐values between 0.05 and 4.17 × 10^−3^ were considered suggestive associations, indicating potential associations.

The core goal of GPA is to estimate the proportion of SNPs (PM) in these models and assess the statistical significance of the overall genetic overlap by LRT. This approach allows us to understand genetic interactions among different traits in a more comprehensive way and provides richer information about the genetic background of the study. The significance level was set at *p* < 4.17 × 10^−3^ (0.05/12) after Bonferroni correction. If the *p*‐value was between 0.05 and 4.17 × 10^−3^, we regarded it as the presence of a suggestive association.

### 2.3 Identification of Pleiotropic Loci and Co‐localization Analysis

In cases where trait pairs exhibited noteworthy genetic correlations or overlaps, this study implemented the PLACO approach to pinpoint potential pleiotropic SNVs (Ray and Chatterjee 2020, Ray et al. 2021). In the study, SNVs with a *p*‐value less than 5 × 10^−8^ in the PLACO analysis were identified as significant pleiotropic variants. To further characterize these loci, Functional Mapping and Annotation of Genetic Associations (FUMA) was employed (Watanabe et al. 2017). We used the default settings for FUMA annotation and used data from European pedigrees in 1000 Genomes Project Phase III as a reference panel. SNPs with *p* < 5 × 10^−8^ and independent of each other at *r^2^* < 0.6 within 1 Mb were defined as independently significant SNPs. Lead SNPs are a subset of independently significant SNPs that are independent of each other at *r^2^* < 0.1. Genomic risk loci are identified by combining LD blocks (< 250 kb) of independent significant SNPs that are closely linked to each other. Top lead SNPs were defined as those with the lowest *p*‐value in a given region. functional annotations, including ANNOVAR category (Wang et al. 2010). Functional annotations, including ANNOVAR category (Ray et al. 2021), Combinatorial Annotation Dependent Deletion (CADD) (Kircher et al. 2014) scoring, and RegulomeDB (Boyle et al. 2012) score, also obtained by FUMA. Bayesian colocalization analysis was then conducted to further identify shared causal variants within each pleiotropic locus. The analysis was based on the coloc method using default priors (p1 = 1 × 10^−4^, p2 = 1 × 10^−4^, and p12 = 1 × 10), with loci having a posterior probability for H4 (PP.H4) greater than 0.7 determined as colocalized loci (Giambartolomei et al. 2014).

### 2.4 Magma‐based Biological Annotation and Functional Enrichment Analysis

Expanding on the PLACO findings, our study delved into the shared biological processes of these pleiotropic motifs. This involved conducting a GenoMic Annotation (MAGMA) analysis at the gene level, focusing on genes situated within or intersecting with pleiotropic motifs, guided by both PLACO outcomes and single‐trait GWAS data (de Leeuw et al. 2015). The research applied MAGMA analysis on PLACO results and single‐trait GWAS data to identify potential pleiotropic genes, with significance thresholds set differently for each. For the MAGMA analysis of PLACO data, a Bonferroni‐corrected *p*‐value of less than 0.05 was deemed significant, whereas for the original single‐trait GWAS in MAGMA analysis, a *p*‐value of less than 0.05 was used.

For gene set functional enrichment analysis of MAGMA outputs, we (https://www.gsea‐msigdb.org/gsea/msigdb/human/collections.jsp) obtained the latest KEGG and GO sets and used the clusterProfiler in RStudio to perform the analysis. Enrichment analysis (Yu et al. 2012) to obtain the results of gene set enrichment. Setting conditions of at least two enriched genes, as well as a *p*‐value of < 0.05 and an FDR of < 0.05, were considered statistically significant.

### 2.5 Pleiotropic Gene Expression Analysis

The Summary data‐based Mendelian Randomization (SMR) software tool was initially developed to implement the SMR & HEIDI method (Zhu et al. 2016). This method integrates data from GWAS and expression quantitative trait loci (eQTL) studies to assess the pleiotropic association between gene expression levels and complex traits of our interest. In terms of LD calculations, we adopted a method based on the 1000 Genomes European reference data (Auton et al. 2015). P‐values were calculated using the BH method, and results were considered significant with a P fdr < 0.05 and heterogeneity HEIDI p > 0.01 as the inclusion criteria (Zhu et al. 2016).

### 2.6 TSMR Analysis

Finally, we performed two‐way Mendelian randomization analyses of 12 paired features between GI and ND to investigate potential causal features. We used an inverse variance weighting method as the primary analysis for our TSMR analyses using the “TwoSampleMR” package in RStudio (version 4.3.0, https://www.r‐project.org/). TSMR analyses (Emdin et al. 2017, Smith and Ebrahim 2003, Haycock et al. 2017) are based on three core assumptions: (1) there is a robust and significant association between Instrumental Variable (IV) and exposure factors; (2) IV is independent of confounders; and (3) IV affects the outcome only by influencing exposure factors and has no other pathways (Davey Smith and Hemani 2014).

To assess the heterogeneity among different Single Nucleotide Polymorphisms (SNP), we used Cochran's *Q* statistic methodology (Hemani et al. 2018). To verify the robustness of the results, we performed a sensitivity analysis. We also applied the MR‐Egger method to detect the presence of pleiotropy in SNPs, measuring directional pleiotropy by the intercept of MR‐Egger regression (Bowden et al. 2015). We chose a Benjamini–Hochberg *p* < 0.05 as the significance threshold.

## 3 Results

### 3.1 Genetic Correlation and Overlap Between GI and ND

Using summary statistics from publicly available genome‐wide association studies, significant genetic overlaps were identified in 12 paired traits. LDSC revealed suggestive associations in two trait pairs, IBD‐PD and GERD‐PD, with GERD‐PD also showing suggestive association in HDL. Notably, the other ten trait pairs were identified as having significant genetic overlap but not genetic correlation, resulting in a final combined set of 12 paired traits for subsequent analysis. The LDSC results were highly consistent with those from High‐definition Likelihood (Supplementary Tables S2–S5) (Figure 2).

Local genetic correlation analysis revealed 28 significant regions, of which 23 were unique significant regions (P_fdr_ < 0.05). The correlation coefficients (rho) of six loci were equal to −1 or 1, indicating strong genetic correlations. Notably, two regions between DVD and PD phenotypes exhibited positive and negative genetic associations, respectively, as detailed in Supplementary Tables S6–S7. Both regions between IBD and PD phenotypes also showed positive correlations, as specifically seen in Supplementary Tables S6–S7. Compared to global genetic correlation, local genetic correlation analysis can more accurately capture genetic links showing mixed effect directions. This means that even if a pair of phenotypes are not significant in global genetic correlation analysis, it is possibly due to a rough balance of positive and negative (opposite effect direction) local genetic correlations. Therefore, even in cases where significant correlations were not detected in LDSC and HDL analyses (P_fdr_ < 0.05), LAVA still identified some significant regions (P_fdr_ < 0.05). Moreover, although only suggestive associations were shown between IBD and PD and GERD and PD in global genetic correlation analysis, local genetic correlation analysis revealed potential associations between these phenotype pairs in multiple regions (*p* < 0.05), and in the IBD and PD phenotype pair, some regions showed significant genetic correlations (P_fdr_ < 0.05), as detailed in Supplementary Tables S6–S7 (Figure 3).

### 3.2 Shared Loci Between GI and ND

Through the PLACO method, the study identified 1457 SNVs (with 1213 being unique) as potential pleiotropic variants across a dozen trait pairs. Concurrently, FUMA successfully pinpointed 66 independent genomic loci with pleiotropic characteristics, covering 47 distinct chromosomal regions. (Supplementary Tables S8‐S9) (Supplementary Figure S1). Pleiotropic regions identified in multiple paired traits, such as 19q13.32 (mapped genes: *APOE*, *CKM*, *PVRL2*, and *PVRL2*:*CTB‐129P6.4*) and 1p31.3 (mapped genes: *PDE4B*, *IL12RB2*, *IL23R*, and *C1orf141*:*IL23R*), along with 2q14.3 (mapped gene: *BIN1*), indicate these sites' broad pleiotropy. Out of the 66 pleiotropic loci identified by FUMA, top SNVs in 58 pairs showed significance in both traits. Twenty‐seven of these pairs (about 47%) demonstrated consistent associations, suggesting that these variants might concurrently influence the risk factors for both GI and mental health conditions. Conversely, the remaining 31 pairs (around 53%) presented inconsistent associations, hinting at the possibility of diverse underlying biological processes (Supplementary Table S10).

The ANNOVAR category annotation revealed that within the 66 indexed SNVs, 33 (50%) were intronic, 15 (23%) intergenic, and only 2 (3%) exonic. Interestingly, these two exonic variants were located at distinct loci and corresponded to two different genes. Specifically, the index SNV rs429358 at 19q13.32 (PLACO *p* = 7.99 × 10^−57^) in *APOE*, a gene associated with various physiological processes including cholesterol metabolism and Alzheimer's disease risk, was significantly associated with AD (original GWAS *p* = 0, recalculated *p* < 2.2 × 10^−16^) but not with DVD GWAS. The index SNV rs34311866 at 4p16.3 (PLACO p = 6.30 × 10^−11^) in *TMEM175* was identified with an exonic variant CADD score > 12.37, rs429358 (CADD score: 12.64; mapped gene: *APOE*), indicating potential deleteriousness. An intronic SNV, rs344816, identified in the GERD‐AD trait pair with a RegulomeDB (RDB) score of 1b, suggests this variant is likely to affect transcription factor binding and is closely related to the regulation of adjacent gene expression.

Subsequent colocalization analysis of the 66 identified potential pleiotropic loci showed that 9 loci (approximately 14%) had a PP.H4 value greater than 0.7 (Table 1). Within these 9 loci, 7 top SNVs were singled out as likely shared causal variants. Surprisingly, the 3q25.1 locus identified as a pleiotropic locus for two trait pairs, IBS and PD (PP.H4 = 0.9343) as well as DVD and PD (PP.H4 = 0.8808), co‐localized between IBS and PD (Top SNP rs9840232, mapped gene *MED12L*) and DVD and PD (Top SNP rs17204437, mapped gene *MED12L*:*P2RY12*) (Supplementary Table S11) (Supplementary Figure S2).

**TABLE 1:** Significant loci identified by FUMA as significant pleiotropic gene loci colocalization results (PP.H4 > 0.7).

Colocalization analysis												
No.	Trait pair	Top SNP	CHR	BP	Locus boundary	Nearest gene	PP.H3	PP.H4	Best causal SNP	SNP.PP.H4	Top SNP	Region
16	DVD‐PD	rs17204437	3	151067007	150800402‐151130197	*MED12L:P2RY12*	0.1164	0.8808	rs17204437	0.0791	rs17204437	3q25.1
23	GERD‐AD	rs2043539	7	12253880	12233848‐12285140	*TMEM106B*	0.0273	0.9669	rs2043539	0.0839	rs2043539	7p21.3
31	GERD‐ALS	rs2059374	7	133094348	132954533‐133643778	*EXOC4*	0.0625	0.9185	rs2059374	0.1317	rs2059374	7q33
38	IBS‐AD	rs3134615	1	40362066	40282818‐40388826	*MYCL*	0.0439	0.8932	rs3134615	0.1987	rs3134615	1p34.2
48	IBS‐PD	rs9840232	3	151052362	150800402‐151130197	*MED12L*	0.0629	0.9343	rs9840232	0.0562	rs9840232	3q25.1
50	IBD‐AD	rs17264332	6	138005515	137959235‐138006504	*RP11‐95M15.1*	0.0113	0.8018	rs17264332	0.2825	rs17264332	6q23.3
51	IBD‐AD	rs2836882	21	40466570	40458508‐40468838	*RPL23AP12*	0.0260	0.9431	rs2836882	0.2066	rs2836882	21q22.2
64	IBD‐PD	rs1736147	21	16813053	16804330‐16841303	*AJ006998.2*	0.0204	0.8009	rs1297261	0.0714	rs1736147	21q21.1
66	IBD‐PD	rs6658353	1	161469054	161463601‐161479745	*FCGR2A*	0.0683	0.8816	rs12139150	0.2796	rs6658353	1q23.3

### 3.3 Pleiotropic Gene Functional Enrichment Analysis

MAGMA analysis identified 82 significant pleiotropic genes (74 unique) from 185 potential pleiotropic genes located in or overlapping with 66 pleiotropic gene loci. Among these, 8 genes showed pleiotropy in two trait pairs, including *CHRNB1*, *MED12L*, *P2RY14*, *GPR87*, *P2RY12*, *BCL7C*, *SNCA*, and *PVRL2* (Supplementary Table S12) (Figure 4).

The gene enrichment analysis revealed 17 significant pathways (*p*‐value < 0.05, P_adjust_ < 0.05). No significant enrichment was observed in KEGG; all pathways were from GO. These pathways mainly relate to signal transduction, amyloid fibril formation and regulation, and lipid metabolism and transport. Notably, four significant pathways, including G protein‐coupled purinergic nucleotide receptor signaling and activity, were identified in both IBS‐PD and DVD‐PD trait pairs. Multiple pathways related to amyloid fibril and protein regulation, as well as cholesterol and lipoprotein particle‐related pathways, were found in the GERD‐AD trait pair (Supplementary Table S13) (Supplementary Figure S3).

### 3.4 Multiple Influences on Gene Expression Levels and Drug Interaction Analysis

We incorporated 74 pleiotropic genes identified by MAGMA analysis into our study and mapped these genes using the eQTLgen gene set, successfully associating them with 57 genes. SMR analysis identified 26 genes with varying correlations to the seven diseases studied (P_fdr_ < 0.05, HEIDI p > 0.01). Among them, *EP300* was related to four diseases. *CHRNB1*, *KNOP1*, *P2RY14*, and *POLR2A* were related to three diseases. *C1orf106*, *FCF1*, *HSD3B7*, *RANGAP1*, *SCAPER*, *SETD1A*, *UBTF*, *VKORC1*, and *ZNF668* were related to two diseases (Supplementary Table S16) (Figure 5).

Additionally, this study retrieved drug‐gene interaction data from the Drug‐Gene Interaction database (DGIdb), identifying 8 genes with interactions with multiple drugs, most of which were inhibitory. Specifically, the *EP300*, *PRKCB*, and *VKORC1* genes demonstrated inhibitory interactions with several drugs. Meanwhile, the *CHRNB1* gene showed interactions with both antagonists and agonists (Supplementary Table S17).

### 3.5 Mendelian Randomization

Bidirectional TSMR was conducted for 12 pairs of GI and ND. Instrumental variable SNPs (*p* < 5×10^−8^) were selected, and linkage disequilibrium was calculated based on the 1000 Genomes European panel using the PLINK clump method, with an *r^2^* < 0.01 (clumping distance = 5000 kb) as the threshold for linkage equilibrium.

In each direction, one significant trait pair was identified. For GERD as the exposure and PD as the outcome, the Mendelian randomization results showed that an increased risk of GERD lowers the risk of PD (P_FDR_ = 0.0105, P_Q_ = 0.3542, P_Egger intercept_ = 0.7779), seemingly validating conclusions drawn from LDSC and HDL analyses (Supplementary Table S14). With ALS as the exposure and DVD as the outcome, increased ALS risk was associated with reduced DVD risk (P_FDR_ = 0.03540, P_Q_ = 0.8478, P_Egger intercept_ = 0.1356) (Supplementary Table S15) (Supplementary Figure S4).

## 4 Discussion

Our study demonstrates significant genetic overlap between GI and ND, with 12 paired traits showing notable pleiotropic associations. Notably, two trait pairs exhibited suggestive correlations in both LDSC and HDL analyses, emphasizing the robustness of these genetic links. Furthermore, LAVA provided a more detailed characterization of the associations between GI and ND and validated the results of the genome‐wide genetic correlations. These similarities indicate the genetic basis of the phenotypic associations. Additionally, 1457 SNVs were identified as potential pleiotropic variants for 12 trait pairs, further confirming the genetic interactions between these diseases. This was particularly evident in shared loci such as 19q13.32 and 1p31.3, which are associated with multiple traits, highlighting their extensive pleiotropic effects.

The presence of both concordant and discordant associations among top SNVs in FUMA‐annotated pleiotropic loci reveals the complex biological mechanisms underpinning these diseases. Exonic variants in the *APOE* and *TMEM175* genes add to this complexity. Previous studies have identified the *APOE* gene as a genetic risk factor for late‐onset AD (Foraker et al. 2015, Yamazaki et al. 2019). The genetic risk factor for PD, *TMEM175*, acts as a proton‐selective channel for proton activation in lysosomes (Hu et al. 2022). *TMEM175*, a genetic risk factor for PD, acts as a proton‐activated, proton‐selective channel in lysosomes (Hemani et al. 2018). Enhanced *TMEM175* function inhibits mitochondrial autophagy, disrupts mitochondrial homeostasis, and increases reactive oxygen species (ROS) production. This creates a positive feedback loop that enhances apoptosis and exacerbates symptoms in the Parkinson's disease mouse model (Qu et al. 2022). Although some studies have shown an association between *TMEM175* and PD, they have not shown an association with intestinal diseases. Our findings therefore provide novel evidence linking *TMEM175* to GI traits, highlighting its potential pleiotropic role in the GBA. Our study unveils a unique association pattern in the original GWAS for the 4p16.3 pleiotropic locus (including *TMEM175*): a risk odds ratio (OR) for GI diseases less than 1, and for ND more than 1. This disparity suggests *TMEM175*'s varied roles in different biological contexts or its differing impacts across physiological systems.

Remarkably, our study found the same pleiotropic regions, 19q13.32, 1p31.3, and 2q14.3, in four trait pairs. Research shows the 19q13.32 region is associated with lifespan (Beekman et al. 2013). Several apolipoprotein genes located at 19q13.32 are linked to cardiovascular metabolic traits and metabolic syndrome's genetic determinants (Yeh et al. 2022). The 1p31.3 locus is a significant risk site for glioma (Melin et al. 2017). Our study is the first to reveal these three loci's pleiotropy in GI and ND.

Colocalization analysis identified nine potential pleiotropic loci, offering deeper insights into the shared causal variants of these diseases. Notably, the 3q25.1 locus exhibited high colocalization between IBS and PD, as well as DVD and PD. The top SNPs, rs9840232 between IBS and PD and rs17204437 between DVD and PD, both mapping to the *MED12L* gene, underscore its potential biological significance in these diseases. Studies have highlighted the relationship between *MED12L* variants and a range of neurological and developmental symptoms, including intellectual disability, developmental delay, speech disorders, autism spectrum disorders, aggressive behavior, and brain abnormalities. GI issues have also been observed in some individuals carrying *MED12L* variants (Nizon et al. 2019). However, its role in ND has been rarely reported. Our study coincidentally finds the 3q25.1 locus's *MED12L* gene significant in both GI and ND, suggesting it as a key biological target that could aid in understanding the pathogenesis and potential treatment strategies of these diseases.

Our gene enrichment analysis, focusing on signal transduction, amyloid fibril formation and regulation, and lipid metabolism and transport, underscores the complexity of cellular processes in disease pathophysiology. Notably, four pathways significant in two trait pairs (IBS‐PD, DVD‐PD) were identified: G protein‐coupled purinergic nucleotide receptor pathways, G protein‐coupled purinergic nucleotide receptor activity, purinergic nucleotide receptor signaling, and nucleotide receptor activity. This finding aligns with current understandings of G protein‐coupled receptors (GPCRs) in various ND and psychiatric diseases, emphasizing their potential as drug targets. Known for their role in signal transduction and ligand diversity, GPCRs are crucial in neural system function and drug development (Wong et al. 2023).

In the GERD‐AD trait pair, our analysis revealed pathways related to amyloid fibril and protein regulation, as well as pathways associated with cholesterol and lipoprotein particles, indicating unique yet overlapping pathophysiological features compared to other studied traits. Cholesterol plays a key role in the pathophysiology of β‐amyloid‐mediated diseases like AD (Rudajev and Novotny 2022). Genes involved in cholesterol metabolism, such as APOE, CLU, and ATP‐binding cassette transporters, including apolipoprotein E, apolipoprotein J, and ATP‐binding cassette transporters, are linked to the pathophysiology of Alzheimer's disease (Reitz 2013). This relationship highlights the importance of cholesterol and its metabolism in amyloid‐related diseases.

Through SMR analysis of blood eQTLs, this study successfully identified several pleiotropic genes, among which the *EP300* gene showed correlations with four diseases. Existing studies have shown that new variants in *EP300* are associated with intellectual disabilities, short stature, and skeletal abnormalities (López et al. 2018, Zimmermann et al. 2007). We found that *EP300* is not only related to AD and PD but also associated with GERD and IBS, the latter of which has been less frequently mentioned in studies. Additionally, the *CHRNB1*, *KNOP1*, *P2RY14*, and *POLR2A* genes each demonstrated associations with three diseases. Furthermore, through the Drug‐Gene Interaction database (DGIdb), we identified the drug GARCINOL, which exhibits an inhibitory interaction with the *EP300* gene. We also identified various drugs associated with the *CHRNB1* gene, including antagonists like GALLAMINE TRIETHIODIDE and RAPACURONIUM BROMIDE, as well as agonists such as SUCCINYLCHOLINE CHLORIDE and DECAMETHONIUM BROMIDE. *EP300* intersects with chromatin regulation and synaptic function, and although p300/CBP inhibitors are being developed in oncology, their repurposing may complement epigenetic strategies for neurodegeneration (Nicosia et al. 2023, Valor et al. 2013, Wang et al. 2025). *PRKCB (PKC‐β)* connects to purinergic GPCR signaling and microglial activation, supporting isoform‐specific PKC modulation as a means to reduce amyloid burden and inflammation (Du et al. 2018, Wu et al. 2025, Zhou et al. 2020). *VKORC1* influences vitamin K–dependent processes in lipid metabolism and oxidative stress, consistent with evidence for vitamin K supplementation in cognition (Popescu and German 2021, Roumeliotis et al. 2024). *CHRNB1*, a nicotinic receptor subunit, relates to cholinergic signaling with established therapeutic relevance (Posadas et al. 2013, Shimohama et al. 2018). Collectively, these genes anchor enriched pathways—purinergic signaling, amyloid/protein aggregation, and cholesterol/lipoprotein transport—to feasible therapeutic strategies, underscoring the translational value of our findings.

Finally, the MR results complement our study by revealing risk associations of GERD with PD and of ALS with DVD. Our MR finding that genetically predicted GERD is associated with lower PD risk may reflect complex gut–brain biology rather than a simple protective effect. Disruption of vagal signaling—shown epidemiologically to lower subsequent PD risk after truncal vagotomy—supports a model in which gastro‐vagal pathways modulate gut→brain *α*‐synuclein propagation; concurrently, GERD‐related alterations in the upper GI environment and downstream gut microbiota can change neuroinflammatory and protein‐aggregation processes implicated in PD (Svensson et al. 2015, Mahbub et al. 2024, Schaeffer et al. 2020). These findings provide new perspectives for a deeper understanding of the genetic basis of these diseases and the development of potential therapeutic strategies.

Furthermore, the GWAS we use, which are based on European populations, exhibit certain limitations. First, genetic variations specific to certain populations might be overlooked because some variants may be more common or related to diseases in other populations. Second, the uniqueness of lifestyle and environmental factors in European populations could affect the relationship between genetic variations and diseases. Additionally, genetic variations and their associations with diseases observed exclusively in European populations may not be applicable to others. Finally, the limitations of this research scope might impact the general applicability of drug development and treatment strategies. In the future, it is crucial to have data from diverse ethnic groups for analysis, as including populations with different ethnic and geographical backgrounds is essential for obtaining a more comprehensive understanding of genetic diversity and more accurate disease association studies.

## 5 Conclusion

In summary, our study has unveiled several pleiotropic gene loci and colocalizations between GI and ND, underscoring the importance of the GBA in the etiology of these diseases. The identification of pleiotropic genes, pathways, and potential drug targets offers valuable insights for future research and treatment.

## Author Contributions

Y.J. led the study design and manuscript writing; Y.Z. assisted with conceptual development; L.M. and C.L. performed data analysis; all authors contributed to manuscript revision and approved the final version.

## Conflicts of Interest

The authors declare no conflicts of interest.

## Funding

This work was supported by the Third Batch of the “Tianshan Talent” High‐Level Medical and Health Personnel Project (Grant Number TSYC202401B119).

## Supporting information




**Supplementary Figures**: brb371029‐sup‐0001‐FigureS1‐S4.docx

**Supplementary Tables**: brb371029‐sup‐0002‐TableS1‐S18.xlsx

**Supplementary Material**: brb371029‐sup‐0003‐SuppMat.jpg

**Supplementary Material**: brb371029‐sup‐0004‐SuppMat.jpg

**Supplementary Material**: brb371029‐sup‐0005‐SuppMat.tif

**Supplementary Material**: brb371029‐sup‐0006‐SuppMat.tif

## Data Availability

This study utilized GWAS data from IEU, GWAS Catalog, PGC database, and the International Parkinson's Disease Genomics Consortium. LDSC: https://github.com/bulik/ldsc. HDL: https://github.com/zhenin/HDL. GPA: https://github.com/dongjunchung/GPA. LAVA: https://github.com/josefin‐werme/LAVA. PLACO: https://github.com/RayDebashree/PLACO. FUMA: https://fuma.ctglab.nl/. MAGMA: https://ctg.cncr.nl/software/magma. SMR: https://yanglab.westlake.edu.cn/software/smr/#Overview. COLOC: https://github.com/chr1swallace/coloc. EQTL: https://eqtlgen.org/cis‐eqtls.html.
