# Supplementary material for: Comprehensive Genome‐Wide Analysis of Shared Genetic Factors in Gastrointestinal and Neurodegenerative Diseases
Source: Brain Behav. 2025 Nov 23;15(11):e71029. doi: 10.1002/brb3.71029 (PMC12641108; doi:10.1002/brb3.71029)
Supplement: Supplementary file 1 — Supplementary Figures: brb371029‐sup‐0001‐FigureS1‐S4.docx [file BRB3-15-e71029-s004.docx]

Figure S1: The Q-Q plot illustrates the expected log10 P-values (on the x-axis) against the observed log10 PPLACO values (on the y-axis). (A)DVD-AD(B)DVD-ALS(C)DVD-PD(D)GERD-AD(E)GERD-ALS(F)GERD-PD(G)IBS-AD(H)IBS-ALS(I)IBS-PD(J)IBD-AD(K)IBD-ALS(L)IBD-PD





Figure S2: A graph showing nine colocalization results for original GWAS trait pairs of gastrointestinal diseases and neurodegenerative diseases, with PP.H4 values exceeding 0.7.(A)3q25.1 for DVD-PD(B)7p21.3 for GERD-AD(C)7q33 for GERD-ALS(D)1p34.2 for IBS-AD(E)3q25.1 for IBS-PD(F)6q23.3 for IBD-AD(G)21q22.2 for IBD-AD(H)21q21.1 for IBD-PD(I)1q23.3 for IBD-PD





Figure S3:GO enrichment analysis results.(A)DVD-PD.(B)GERD-AD(C)IBD-AD(D)IBS-PD


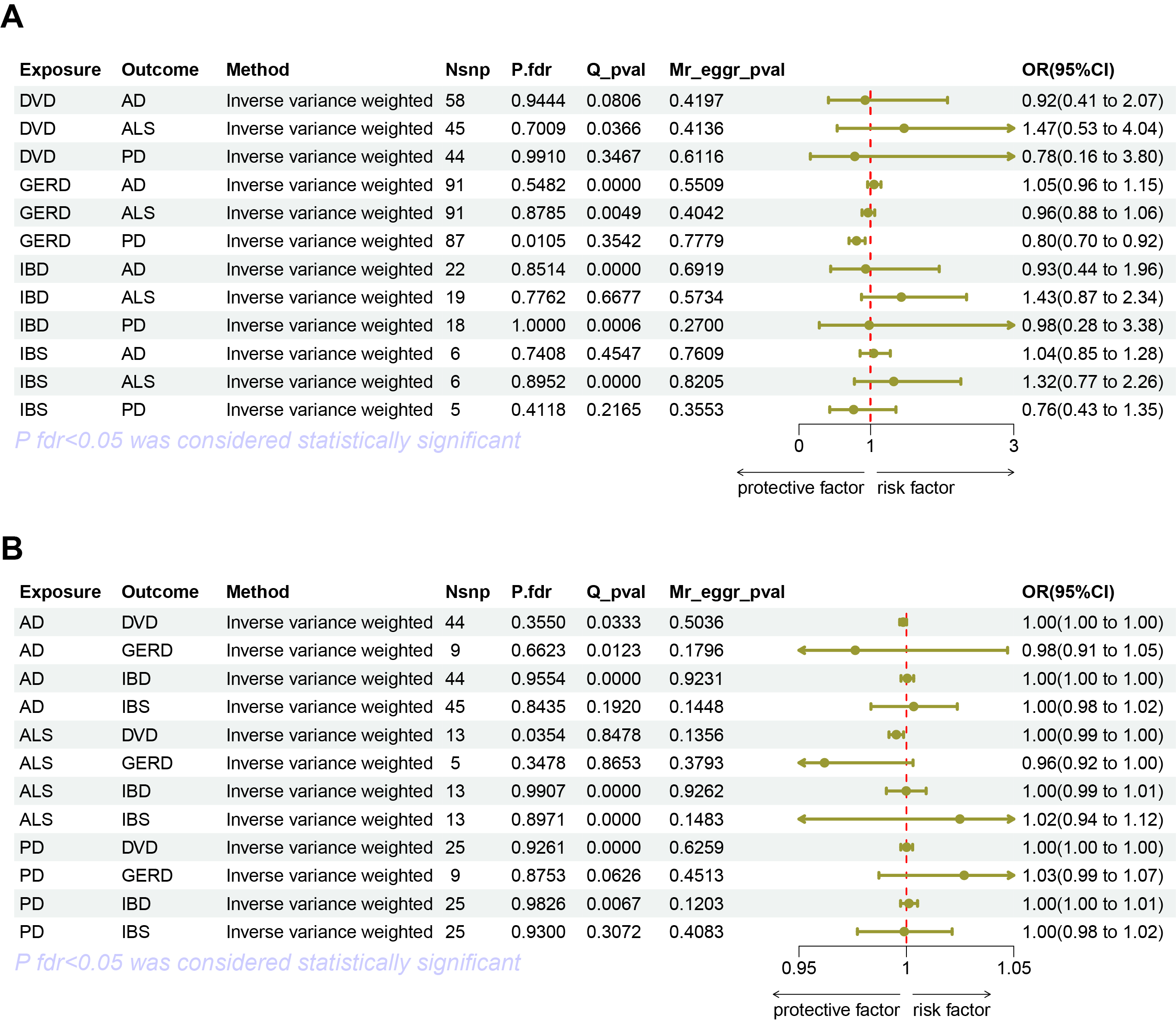


Figure S4:MR Results.(A) MR results with GI as exposure and ND diseases as outcomes.(B) MR results with ND as exposure and GI as outcomes.
