# Supplementary figures and images for: Comprehensive Genome‐Wide Analysis of Shared Genetic Factors in Gastrointestinal and Neurodegenerative Diseases
Source: Brain Behav. 2025 Nov 23;15(11):e71029. doi: 10.1002/brb3.71029 (PMC12641108; doi:10.1002/brb3.71029)

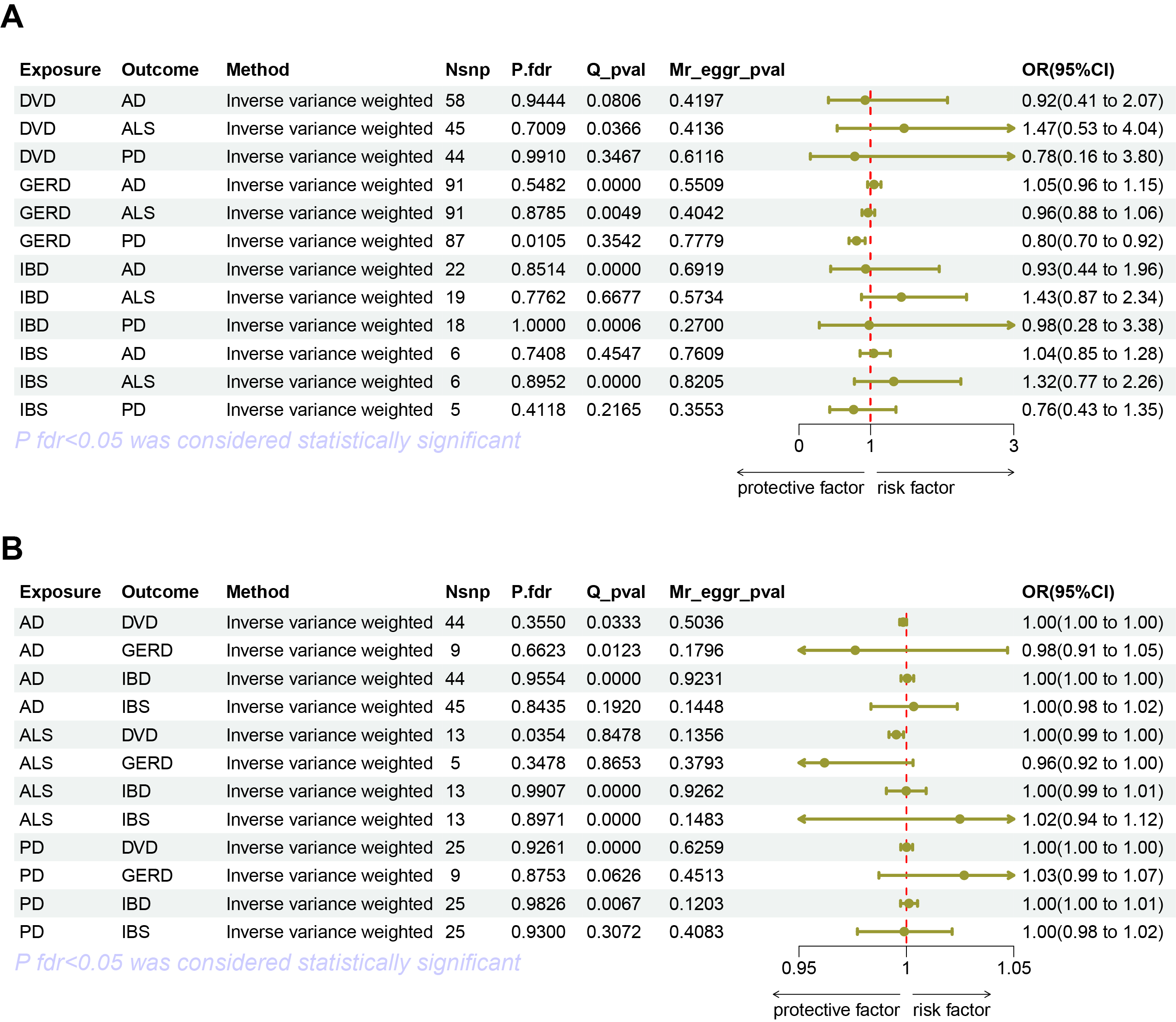

Supplement: Supplementary file 6 — Supplementary Material: brb371029‐sup‐0006‐SuppMat.tif [file BRB3-15-e71029-s005.tif]
